# Integrative analysis of a novel snoRNA-based prognostic signature in patients with breast cancer

**DOI:** 10.3389/fonc.2026.1779697

**Published:** 2026-04-13

**Authors:** Yi Xun, Xia-hui Li, Wen-tao Xiao, Jun-yan He

**Affiliations:** 1 Cancer Research Institute, The First Affiliated Hospital, Hengyang Medical School, University of South China, Hengyang, China; 2 Department of Oncology, The First Affiliated Hospital, Hengyang Medical School, University of South China, Hengyang, China; 3 Department of Radiation Oncology, Affiliated Hospital of Nantong University, Nantong University, Nantong, China

**Keywords:** bioinformatics analysis, biomarker, breast cancer, prognostic signature, snoRNA

## Abstract

**Background:**

Breast cancer (BC) is the most commonly diagnosed malignancy in women worldwide. Prognostic heterogeneity driven by molecular subtypes and tumor microenvironment underscores the need for novel biomarkers. Small nucleolar RNAs (snoRNAs) have emerged as potential regulators in cancer biology, but their prognostic value in BC remains unclear.

**Methods:**

We analyzed snoRNA expression profiles of 1, 025 BC patients from TCGA database. Univariate Cox, least absolute shrinkage and selection operator (LASSO), and multivariate Cox regression analyses were used to construct an 8-snoRNA prognostic signature. The model was validated in testing and entire cohorts. Gene set enrichment analysis (GSEA) and immune infiltration analyses were conducted to explore molecular mechanisms. SNORA11 was functionally validated through *in vitro* assays and transcriptome sequencing.

**Results:**

We identified an 8-snoRNA signature (SNORD114-29, SNORA5A, SNORA54, SNORA7B, SNORA9, SNORA11, SCARNA3, and SNORD64) that effectively stratified patients into high- and low-risk groups, with the high-risk group exhibiting significantly poorer survival outcomes. The prognostic model demonstrated good predictive performance, with AUC values of 0.775, 0.679, and 0.718 for 1-, 3-, and 5-year overall survival (OS) in the testing cohort, respectively, and comparable performance in the entire cohort (0.758, 0.708, and 0.715). The model correlated with aggressive clinical features such as tumor stage, subtype, and tumor mutation burden (TMB). GSEA analysis indicated that high-risk patients showed enrichment of proliferative pathways and suppression of immune signaling. Immune infiltration analysis revealed reduced anti-tumor immune cell infiltration in the high-risk group. Overexpression of SNORA11 enhanced BC cell proliferation, migration, and invasion, and transcriptomic analysis further revealed that SNORA11 overexpression is associated with enhanced proliferative signaling and suppressed immune-related pathways.

**Conclusions:**

We established a novel snoRNA-based prognostic model with strong predictive power and biological relevance in BC. SNORA11 was identified as a potential oncogenic snoRNA, offering new insights into BC progression and potential therapeutic targets.

## Introduction

Breast cancer (BC) is the most frequently diagnosed malignancy and the leading cause of cancer-related mortality among women globally. According to recent global cancer statistics, more than 2.3 million new cases and approximately 665, 000 deaths occur annually, underscoring its significant public health burden (1). In the United States alone, an estimated 316, 950 new cases and 42, 170 deaths are expected in 2024 (2). Despite substantial progress in early detection, molecular subtyping, and multimodal treatments—including surgery, chemotherapy, endocrine therapy, targeted therapy, and immunotherapy—clinical outcomes remain highly variable (3–5). This heterogeneity is attributed to diverse molecular subtypes, complex tumor microenvironments, and variable therapeutic responses, which complicate accurate risk stratification (6, 7). Consequently, identifying reliable prognostic biomarkers and developing robust predictive models has become increasingly critical to facilitate personalized treatment approaches and enhance survival outcomes in BC patients.

Small nucleolar RNAs (snoRNAs) are a conserved class of small non-coding RNAs, typically 60–300 nucleotides in length, primarily localized in the nucleolus (8). Traditionally, snoRNAs are classified into C/D box and H/ACA box families, guiding 2’-O-methylation and pseudouridylation of ribosomal and small nuclear RNAs, respectively. Beyond their canonical roles in RNA modification, recent studies have revealed non-canonical functions of snoRNAs in alternative splicing, chromatin remodeling, and regulation of gene expression (9). Growing evidence indicates that dysregulated snoRNAs are involved in tumorigenesis, influencing cell proliferation, invasion, immune modulation, and therapy resistance (10, 11). In BC, several snoRNAs, such as SNORA71A, SNORD50A, and U50A, have been linked to tumor progression, metastasis, or prognosis (12–15). However, the prognostic value of snoRNAs in BC remains poorly defined. Therefore, a systematic analysis of snoRNA expression is warranted to uncover novel prognostic markers and provide insights into BC biology.

Given the oncogenic roles of snoRNAs and their potential as molecular biomarkers, we hypothesize that specific snoRNA expression profiles may serve as effective predictors of clinical outcomes in BC. Nevertheless, to date, no comprehensive snoRNA-based prognostic model has been established for BC. Furthermore, the relationships between snoRNA expression, the tumor immune microenvironment, molecular subtypes, and treatment response remain inadequately characterized. With the availability of large-scale transcriptomic datasets and advanced computational tools, it is now feasible to conduct a systematic investigation of the prognostic relevance of snoRNAs in BC. Developing a robust snoRNA-based prognostic signature could improve risk stratification and provide novel insights into the molecular mechanisms underlying BC progression.

In this study, we utilized snoRNA expression profiles from The Cancer Genome Atlas (TCGA), curated through the SNORic database, to perform univariate Cox regression analysis and identify prognostically relevant snoRNAs in BC. An eight-snoRNA prognostic signature was then developed using least absolute shrinkage and selection operator (LASSO) and multivariate Cox regression analyses. The model showed strong predictive performance, effectively stratifying patients into high- and low-risk groups based on overall survival (OS). Importantly, its prognostic value was further validated in internal cohort. To investigate the biological relevance of the signature, we performed gene set enrichment analysis (GSEA) and immune cell infiltration analysis. Functional assays in BC cell lines confirmed the oncogenic role of SNORA11. Transcriptome sequencing further indicated that SNORA11 may promote tumor progression by activating cell proliferation pathways and suppressing immune-related responses. Collectively, these findings underscore the prognostic potential of snoRNA-based signatures and lay the groundwork for future mechanistic and translational studies.

## Materials and methods

### Data acquisition

**Figure 1:**
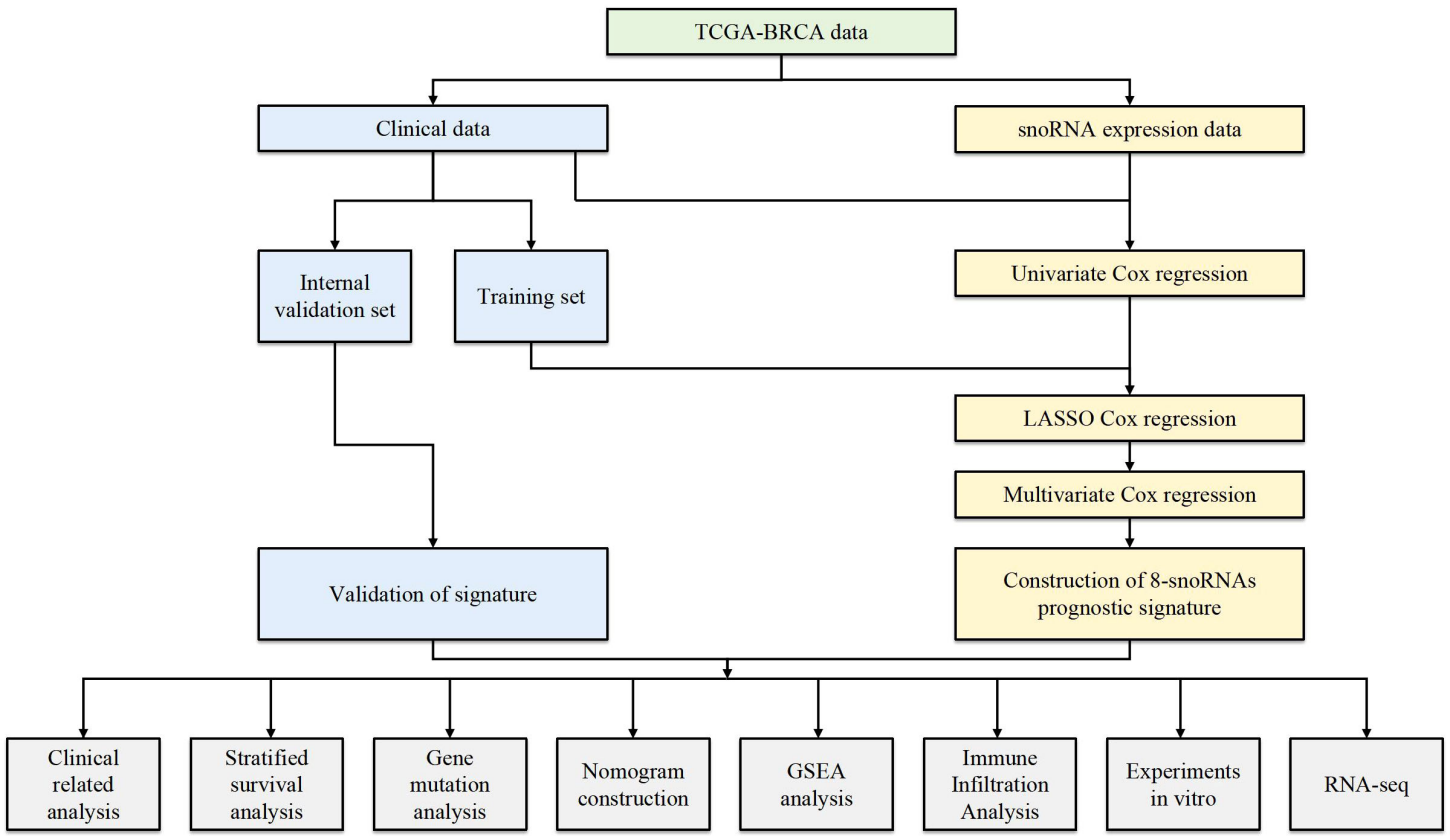
The workflow of the study.

The mRNA expression profiles and clinical data of breast invasive carcinoma (BRCA) patients were retrieved from The Cancer Genome Atlas (TCGA, https://portal.gdc.cancer.gov/) (16). snoRNA expression data for BRCA patients, provided in Reads Per Kilobase per Million (RPKM) format, were downloaded from the SNORic database (https://github.com/chunjie-sam-liu/SNORic) (17). While TPM or raw counts are preferred in some studies, RPKM has been commonly used for snoRNA analyses and was selected for this study due to its widespread availability in the SNORic database. The snoRNA expression values were log-transformed [log(RPKM + 1)] prior to downstream analysis. To minimize bias, patients with missing survival data or an OS time of less than 30 days were excluded, resulting in a final cohort of 1, 025 patients. To ensure data reliability, snoRNAs expressed in more than 75% of the total patient cohort were defined as effectively expressed snoRNAs in BRCA. In total, 1, 025 patient samples and 467 effectively expressed snoRNAs were included in the subsequent analyses. An overview of the study design and analytical workflow is illustrated in **Figure 1**.

### Construction and validation of a prognostic signature based on snoRNAs

A total of 1, 025 BRCA patients were randomly divided into training and testing cohorts at a 1:1 ratio. In the training cohort, univariate Cox regression analysis was initially conducted to identify snoRNAs significantly associated with OS in BRCA. The glmnet R package (https://CRAN.R-project.org/package=glmnet) was then used to perform LASSO Cox regression analysis to select the most prognostically relevant snoRNAs. The optimal penalty parameter (λ) was determined using 10-fold cross-validation, and the value corresponding to the minimum cross-validated partial likelihood deviance (lambda.min) was selected. As implemented in the glmnet package, predictor variables were automatically standardized prior to model fitting. The selected snoRNAs were further refined using stepwise multivariate Cox regression analysis to construct a robust prognostic model. The risk score for each patient was calculated using the following formula: Risk Score = (expression of snoRNA_1_ × coefficient_1_) + (expression of snoRNA_2_ × coefficient_2_) +… + (expression of snoRNA_n_ × coefficient_n_). Patients were categorized into high-risk and low-risk cohorts based on the median risk score. To evaluate OS between these groups, Kaplan-Meier survival analysis was executed, with statistical significance determined via the log-rank test. A time-dependent receiver operating characteristic (ROC) curve analysis was performed utilizing the timeROC R package (https://CRAN.R-project.org/package=timeROC), with survival endpoints set at 1, 3, and 5 years to assess the model’s predictive capability. The area under the ROC curve (AUC), in conjunction with sensitivity and specificity metrics, was employed to gauge predictive accuracy. To further substantiate the robustness and generalizability of the snoRNA-based signature, both Kaplan-Meier and ROC analyses were carried out in the testing cohort, as well as across the entire study population.

### Association between snoRNAs signature and clinicopathological characteristics

The relationship between the signature and a range of clinicopathological characteristics was illustrated through the utilization of R packages, specifically ggplot2 (https://CRAN.R-project.org/package=ggplot2), ggpubr (https://CRAN.R-project.org/package=ggpubr), and pheatmap (https://CRAN.R-project.org/package=pheatmap). Furthermore, stratified OS analyses were performed employing the R packages survival (https://CRAN.R-project.org/package=survival) and survminer (https://CRAN.R-project.org/package=survminer) to assess the prognostic significance of the snoRNA signature across distinct clinical subgroups. These subgroup analyses included stratification by age, pathologic stage, and molecular subtype, thereby assessing the consistency and robustness of the prognostic model across clinically relevant categories.

### Genomic alterations and tumor mutation burden analysis

Analysis of genomic alterations and tumor mutation burden (TMB) was conducted by evaluating somatic mutation profiles within both high-risk and low-risk patients. This analysis utilized the R packages maftools (https://bioconductor.org/packages/maftools/), ggplot2, and forestplot (https://CRAN.R-project.org/package=forestplot).

### Nomogram construction

To identify independent prognostic determinants, both univariate and multivariate Cox regression analyses were performed utilizing the survival R package. A prognostic nomogram was developed through the rms R package (https://CRAN.R-project.org/package=rms), which integrated prognostic variables such as the pathologic T, N, and M stages, the status of triple-negative breast cancer (TNBC), as well as the risk score. The nomogram’s predictive accuracy was assessed using calibration curves, which compare predicted probabilities with observed outcomes to evaluate model reliability. Predictive performance was further evaluated by calculating the concordance index (C-index) and the ROC analysis, using the riskRegression (https://CRAN.R-project.org/package=riskRegression) and timeROC R packages.

### GSEA

GSEA was performed to identify significantly enriched hallmark pathways between the high-risk and low-risk groups. BRCA patients in the TCGA cohort were stratified into high-risk and low-risk groups based on the median risk score. GSEA was performed using the clusterProfiler R package(https://bioconductor.org/packages/clusterProfiler/). Enrichment results were evaluated based on the Normalized Enrichment Score (NES) together with nominal p-values and adjusted p-values. Pathways with adjusted p-value < 0.05 were considered significantly enriched. For visualization, the top enriched pathways were displayed according to enrichment ranking.

### Immune infiltration analysis

The ESTIMATE R package (https://CRAN.R-project.org/package=tidyestimate) was used to evaluate immune infiltration and the tumor microenvironment by calculating immune scores, stromal scores, and the composite ESTIMATE score. To further characterize the immune landscape, single-sample gene set enrichment analysis (ssGSEA) was performed using the GSVA R package (https://bioconductor.org/packages/GSVA/) to quantify the infiltration levels of 28 tumor-infiltrating immune cell types. Following this, both Spearman and Pearson correlation analyses were performed to evaluate the relationships between the levels of immune cell infiltration and the prognostic risk scores.

### Cell culture

The BC cell lines MDA-MB-231 and MDA-MB-436 were sourced from the Cell Bank of the Chinese Academy of Sciences, located in Shanghai, China. The cells were cultivated in Dulbecco’s Modified Eagle Medium (DMEM; Gibco, USA), enriched with 10% fetal bovine serum (FBS) and 1% penicillin-streptomycin solution (P1400; Solarbio, China). All cultures were sustained at a temperature of 37 °C within a humidified incubator that maintained an atmosphere of 5% CO_2_.

### RNA isolation and qRT-PCR

Total RNA was extracted from harvested cells using TRIzol reagent (Invitrogen, Thermo Fisher Scientific, USA) according to the manufacturer’s instructions, and subsequently reverse transcribed into cDNA using the RevertAid First Strand cDNA Synthesis Kit (R333-C1, Vazyme, China). Quantitative real-time PCR (qRT-PCR) was conducted using SYBR Premix DimerEraser (Takara, Japan). For SNORA11 expression analysis, U6 was used as the internal control, and Relative gene expression was calculated using the 2^–ΔΔCq method. The following primers were used: SNORA11 forward: 5′-CCCAACAGGAATCTGGGGTC-3′, SNORA11 reverse: 5′-GTGTTGGGGGACGTTTGTTC-3′, U6 forward: 5′-CTCGCTTCGGCAGCACA-3′, U6 reverse: 5′-AACGCTTCACGAATTTGCGT-3′.

### SNORA11 overexpression

Plasmids containing the SNORA11 expression cassette and the corresponding control vector were purchased from GenePharma (Suzhou, China). For plasmid transfection, exponentially growing cells were seeded in six-well plates. When the cells reached 70-80% confluence, they were transfected with either the SNORA11 plasmid or the control vector using Lipofectamine 3000 (Invitrogen, USA) according to the manufacturer’s protocol. Transfected cells were incubated for 48 hours before subsequent analyses.

### CCK-8 assay

Cell viability was assessed using the Cell Counting Kit-8 (CCK-8) assay. SNORA11-transfected MDA-MB-231 and MDA-MB-436 cells were resuspended in complete culture medium and seeded into 96-well plates at a density of 1, 000 cells per well. To monitor proliferation dynamics, cells were incubated for the indicated time periods. Subsequently, 10 µL of CCK-8 solution (BMU106, Abbkine, China) was added to each well, followed by a 2-hour incubation at 37°C in a humidified incubator. The number of viable cells was determined by measuring absorbance at 450 nm using a spectrophotometer.

### Colony formation assay

SNORA11-transfected MDA-MB-231 and MDA-MB-436 cells were resuspended in complete culture medium and seeded into 6-well plates at a density of 1, 000 cells per well. To evaluate the long-term proliferative capacity, cells were incubated for 14 days at 37°C in a humidified incubator with 5% CO_2_. After incubation, cells were fixed with 4% paraformaldehyde (P1110, Solarbio, China) and stained with 0.1% crystal violet (G1062, Solarbio, China). The total area covered by the colonies was captured using a camera, and the number of colonies was counted using ImageJ software.

### Wound healing assay

Cells were seeded evenly into 6-well plates and grown to approximately 90% confluence. A sterile 20µL pipette tip was then used to create linear scratches across the cell monolayer. The cells were washed to remove debris and cultured in serum-free medium at 37°C. Images of the wound area were captured at defined time intervals using a phase-contrast microscope. Wound closure was quantified by analyzing the captured images using ImageJ software.

### Transwell assay

For the purposes of conducting both migration and invasion assays, cell culture inserts featuring transparent PET membranes with an 8 µm pore size in a 24-well format (Nest, NY, China) were employed. In the migration assay, 5 × 10^4^ BC cells were suspended in serum-free DMEM and subsequently introduced into the upper chambers of the inserts. Meanwhile, the lower chambers were filled with 500 µL of a complete medium enriched with FBS, serving as a chemoattractant. In the case of the invasion assay, the same quantity of BC cells (5 × 10^4^) was placed into the upper chambers of inserts that had been coated with Matrigel (#354234, Corning, USA). Following a 48-hour incubation period at 37 °C, the cells were rinsed with PBS, fixed using methanol, and stained with a 0.5% crystal violet solution. Cells that had neither migrated nor invaded and remained on the upper surface of the membrane were eliminated with a cotton swab. Three randomly selected fields per insert were captured using a microscope, and the quantification of the migrated or invaded cells was performed utilizing ImageJ software.

### RNA sequencing analysis

Total RNA was extracted from negative control (NC) and SNORA11 overexpressed (OE-SNORA11) MDA-MB-231 cells for RNA sequencing (RNA-seq), with five biological replicates per group. RNA-seq library preparation and next-generation sequencing were conducted by Tsingke Biotechnology (China). Differentially expressed genes (DEGs) were identified using an adjusted p-value < 0.05 and a log2(fold change) > 1 or < –1. Gene Ontology (GO) enrichment analysis was performed using the R packages clusterProfiler, org.Hs.eg.db (https://bioconductor.org/packages/org.Hs.eg.db/), and enrichplot (https://bioconductor.org/packages/enrichplot/). GSEA was performed to identify enriched hallmark pathways between NC and OE-SNORA11 groups. Analyses were conducted using the clusterProfiler R package, with pathways considered significantly enriched at an adjusted p-value < 0.05.

### Statistical analysis

Statistical analyses were performed using R software (version 4.2.2), with statistical significance defined as p < 0.05. GraphPad Prism (version 9.5) was used for the analysis and visualization of experimental data. To ensure result reliability, each experimental group included at least three independent biological replicates. Differences between groups were evaluated using a two-tailed student’s t-test. Statistical significance was indicated as follows: *P < 0.05, **P < 0.01, ***P < 0.001, ****P < 0.0001.

## Results

### Construction of 8-snoRNA prognostic signature for BRCA patients

**Figure 2:**
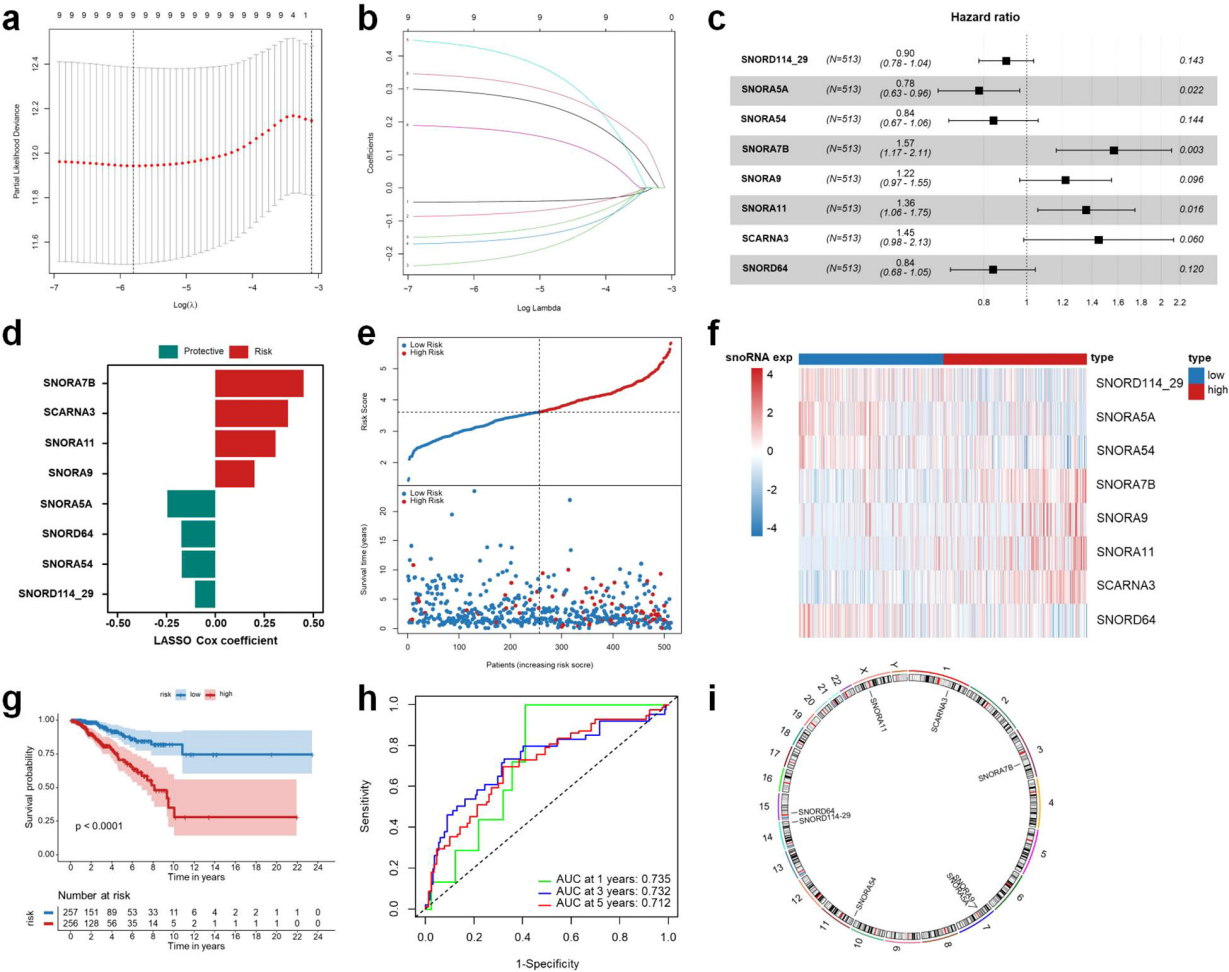
Construction of an 8-snoRNA prognostic signature for BRCA patients. LASSO Cox regression analysis for feature selection of prognostic snoRNAs **(a, b)**. Multivariate Cox regression analysis of the identified prognostic snoRNAs **(c)**. Coefficients of the eight snoRNAs included in the final multivariate Cox regression model **(d)**. Risk score distribution (top) and survival status of BRCA patients (bottom) **(e)**. Heatmap showing the expression levels of the eight snoRNAs in high- and low-risk groups **(f)**. Kaplan-Meier survival curves comparing overall survival (OS) between high- and low-risk groups **(g)**. Time-dependent ROC curves evaluating the predictive accuracy of the 8-snoRNA signature for 1-, 3-, and 5-year OS **(h)**. Genomic locations of the eight prognostic snoRNAs on human chromosomes **(i)**. BRCA, breast invasive carcinoma; LASSO, least absolute shrinkage and selection operator; OS, overall survival; ROC, receiver operating characteristic.

First, all cases in the TCGA-BRCA dataset were randomly divided into a training cohort (n=513) and a testing cohort (n=512). Subsequently, key genes were selected using univariate Cox regression analysis and LASSO Cox regression analysis, resulting in 10 snoRNAs: SNORD114-26 (ENSG00000200413), SNORD114-29 (ENSG00000201689), SNORA5A (ENSG00000206838), SNORA54 (ENSG00000207008), SNORA7B (ENSG00000207088), SNORA9 (ENSG00000199282), SNORA11 (ENSG00000221750), SCARNA3 (ENSG00000252906), SNORD64 (ENSG00000276610), and SNORD116-24 (ENSG00000207279). Further optimization using multivariate Cox regression analysis led to the final selection of eight snoRNAs: SNORD114-29, SNORA5A, SNORA54, SNORA7B, SNORA9, SNORA11, SCARNA3, and SNORD64 (**Figures 2a-c**). A risk score model was developed based on these eight snoRNAs, and its calculation formula is as follows: Risk Score = 0.4503700 × SNORA7B + 0.3713122 × SCARNA3 + 0.3069966 × SNORA11 + 0.2008002 × SNORA9 + (-0.1051736) × SNORD114-29 + (-0.1711728) × SNORA54 + (-0.1737142) × SNORD64 + (-0.2457859) × SNORA5A (**Figure 2d**). Patients were categorized into high-risk and low-risk cohorts based on the median risk score. The risk score distribution, survival duration, and patient classification within these two groups are illustrated in **Figure 2e**, while **Figure 2f** presents the expression profiles of snoRNAs across the different risk categories. As indicated in **Figure 2g**, survival analysis demonstrated that patients classified as high-risk experienced markedly inferior survival rates in comparison to those in the low-risk category, underscoring the prognostic significance of the model. These findings indicate that the identified snoRNA signature may capture key biological characteristics associated with breast cancer progression and patient prognosis. Additionally, the model’s robustness was substantiated through ROC curve analysis, which produced AUC values of 0.735, 0.732, and 0.712 for overall survival at 1, 3, and 5 years, respectively (**Figure 2h**), thereby confirming its substantial predictive accuracy. The chromosomal locations of the eight snoRNAs are depicted in **Figure 2i**.

### Validation of 8-snoRNA prognostic signature

**Figure 3:**
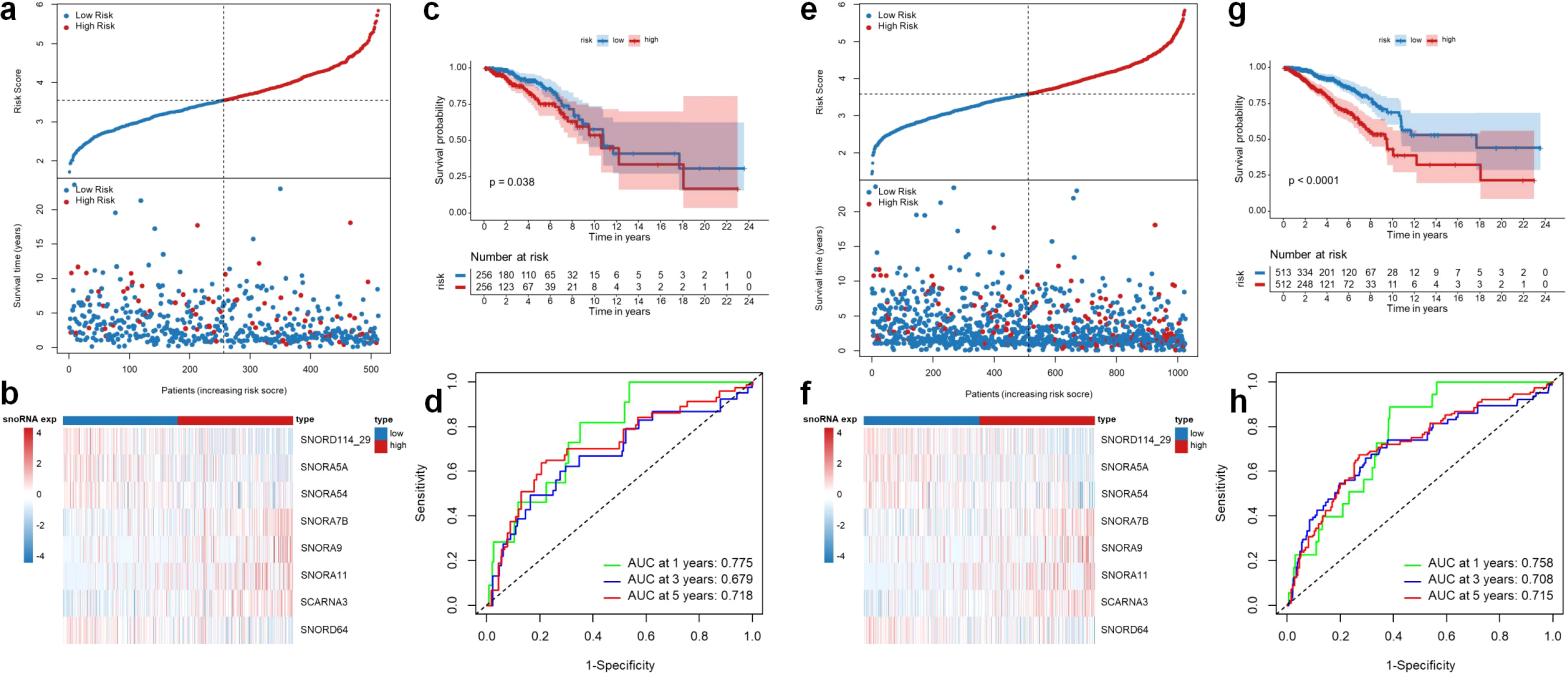
Validation of the 8-snoRNA prognostic signature in the TCGA-test and TCGA-entire cohorts. Risk score distribution (top), survival status (bottom), and snoRNA expression heatmap in the TCGA-test cohort **(a, b)**. Kaplan-Meier survival curves comparing OS between high- and low-risk groups in the TCGA-test cohort **(c)**. Time-dependent ROC curves evaluating the predictive performance of the 8-snoRNA signature for 1-, 3-, and 5-year OS in the TCGA-test cohort **(d)**. Risk score distribution (top), survival status (bottom), and snoRNA expression heatmap in the TCGA-entire cohort **(e, f)**. Kaplan-Meier survival curves comparing OS between high- and low-risk groups in the TCGA-entire cohort **(g)**. Time-dependent ROC curves evaluating the predictive performance of the 8-snoRNA signature for 1-, 3-, and 5-year OS in the TCGA-entire cohort **(h)**. TCGA, The Cancer Genome Atlas; OS, overall survival; ROC, receiver operating characteristic.

To verify the ability of the 8-snoRNA prognostic signature to predict the prognosis of BRCA patients, we calculated the risk score of all patients in the validation set (TCGA-test and TCGA-entire) by the prognostic signature and divided them into high- and low-risk groups based on the median risk score. In the TCGA-test cohort, the expression profiles of the eight snoRNAs, corresponding risk scores, and patient survival status distributions are illustrated in **Figures 3a, b**. Kaplan-Meier survival analysis (**Figure 3c**) revealed that patients in the low-risk group exhibited significantly improved OS compared to those in the high-risk group, consistent with the findings observed in the training cohort. Furthermore, ROC analysis indicated that the AUC values remained high, which yielded AUC values of 0.775, 0.679, and 0.718 for 1-year, 3-year, and 5-year OS, respectively (**Figure 3d**). Similarly, in the TCGA-entire cohort, the distribution of snoRNA expression levels, risk scores, and survival outcomes are presented in **Figures 3e, f**. Kaplan-Meier analysis again demonstrated a significantly favorable prognosis in the low-risk group compared to the high-risk group (**Figure 3g**). The prognostic signature maintained robust predictive performance, with AUC values of 0.758, 0.708, and 0.715 for 1-, 3-, and 5-year OS, respectively (**Figure 3h**). Collectively, these results confirm the reliability and robustness of the 8-snoRNA signature as an independent prognostic indicator for BRCA patients across multiple validation cohorts. This consistency across different cohorts further supports the stability and general applicability of the prognostic model.

### Association between 8-snoRNA signature and clinicopathological characteristics

**Figure 4:**
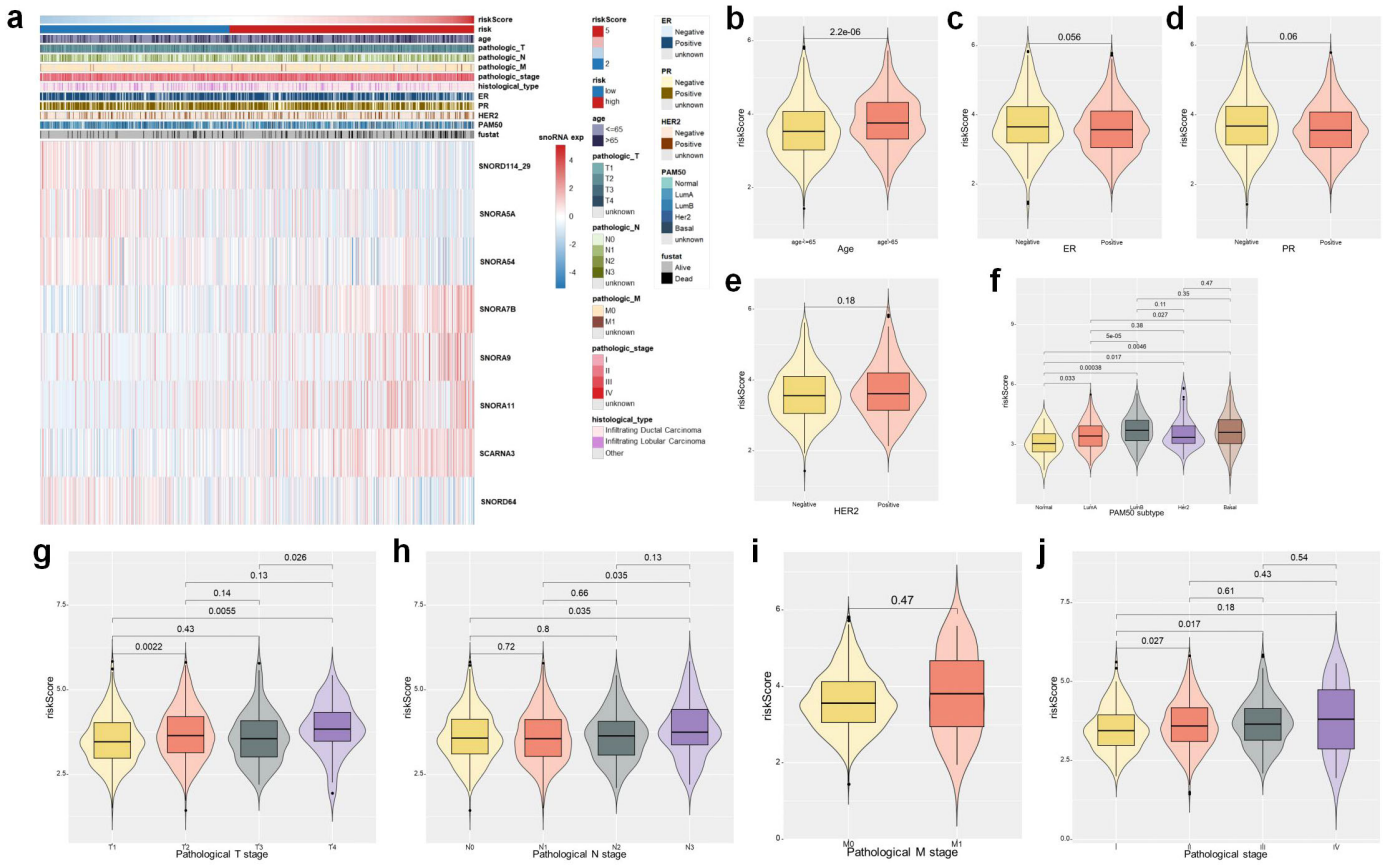
Association between the 8-snoRNA prognostic signature and clinicopathological characteristics in BRCA patients. Heatmap showing the expression patterns of the eight snoRNAs and their correlation with clinicopathological features including age, pathological stage, histological type, hormone receptor status (ER, PR, HER2), and PAM50 subtype **(a)**. Violin plots showing the distribution of risk scores across different clinical subgroups **(b-j)**: Age **(b)**; ER status **(c)**; PR status **(d)**; HER2 status **(e)**; PAM50 molecular subtypes **(f)**; Pathological T stage **(g)**; Pathological N stage **(h)**; Pathological M stage **(i)**; Pathological stage **(j)**. BRCA, breast invasive carcinoma; ER, estrogen receptor; PR, progesterone receptor; HER2, human epidermal growth factor receptor 2; PAM50, prediction analysis of microarray 50.

**Figure 5:**
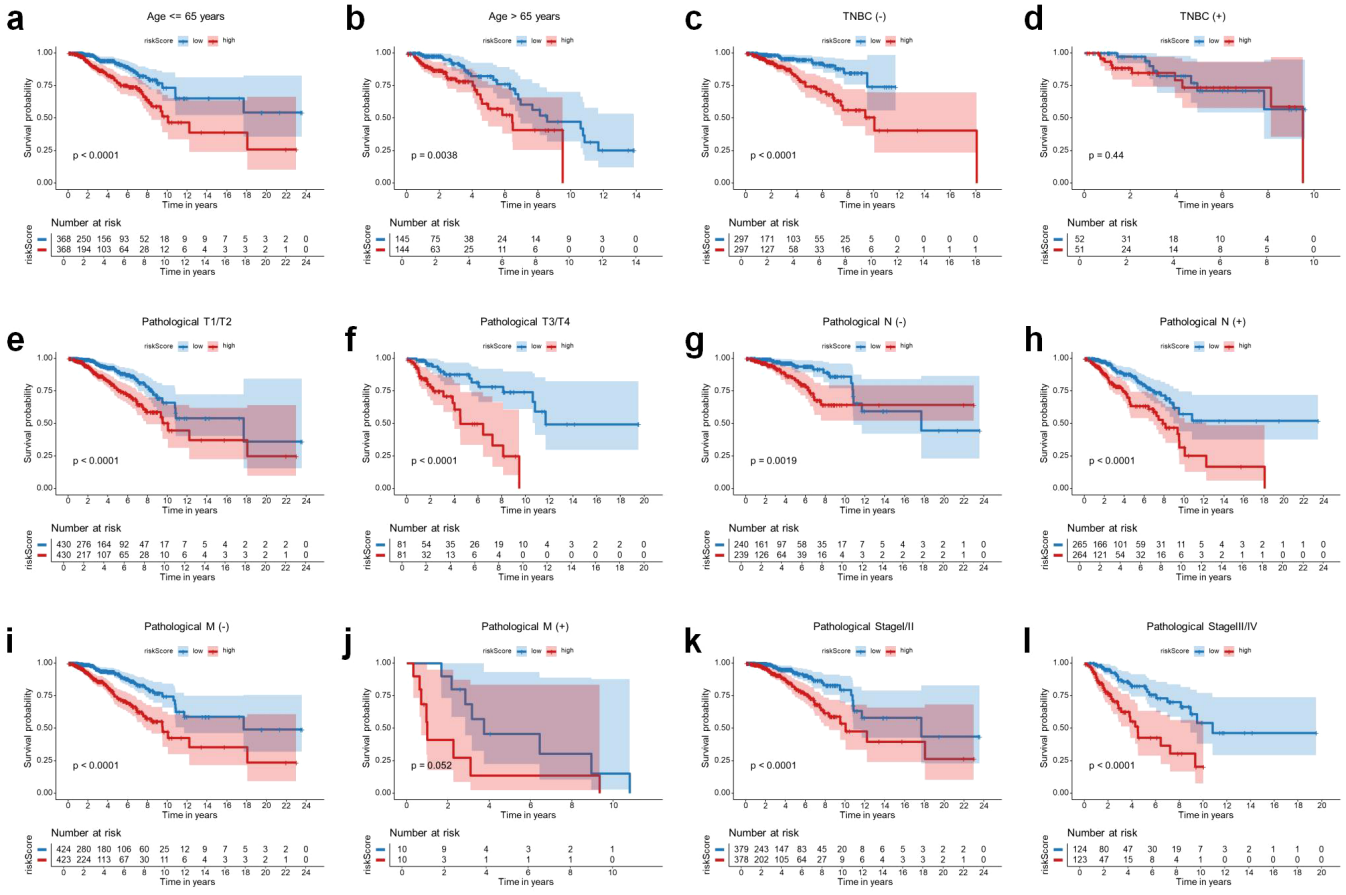
Stratified survival analysis of the 8-snoRNA prognostic signature across clinical subgroups. Kaplan-Meier survival analysis for patients stratified by age **(a, b)**, TNBC status **(c, d)**, pathological T stage **(e, f)**, pathological N stage **(g, h)**, pathological M stage **(I, j)**, pathological stage **(k, l)**. TNBC, triple negative breast cancer.

In order to assess the clinical relevance of the 8-snoRNA signature, we performed an extensive analysis correlating clinical data. The results, illustrated in heatmaps and box plots (**Figures 4a-j**), revealed notable correlations between the 8-snoRNA signature and a range of clinicopathological characteristics. Patients aged > 65 years exhibited significantly higher risks scores compared to those <= 65 years (p = 2.2e−06, **Figure 4b**). Patients with luminal B and basal-like subtypes exhibited significantly higher risk scores compared to those with normal-like and luminal A subtypes (**Figure 4f**). This finding suggests that the 8-snoRNA signature is associated with molecular subtype heterogeneity and may be particularly relevant to more aggressive breast cancer subtypes. Patients with pathological T4 had significantly higher risk scores compared to those with pathological T1 (p = 0.0022) and T2 (p = 0.0055) (**Figure 4g**). Patients with N3 disease exhibited significantly higher risk scores compared to N0 (p = 0.035) and N1 (p = 0.035) groups (**Figure 4h**). An increasing trend of risk score was observed with higher pathological stages (**Figure 4j**), patients with pathological stage IV disease had significantly elevated scores compared to those with stage I (p = 0.027) and stage II (p = 0.017). These results suggest that the risk score is closely associated with tumor progression and aggressive clinical features, supporting its potential utility in prognostic stratification of BC patients. The stratified analysis additionally demonstrated that within various subgroups categorized by age, molecular classification, and pathological stage, patients with elevated risk scores uniformly displayed inferior OS outcomes (**Figures 5a-l**). Notably, the prognostic significance of this signature persisted across multiple clinical subgroups, underscoring its robustness.

### Genomic alterations and TMB analysis

**Figure 6:**
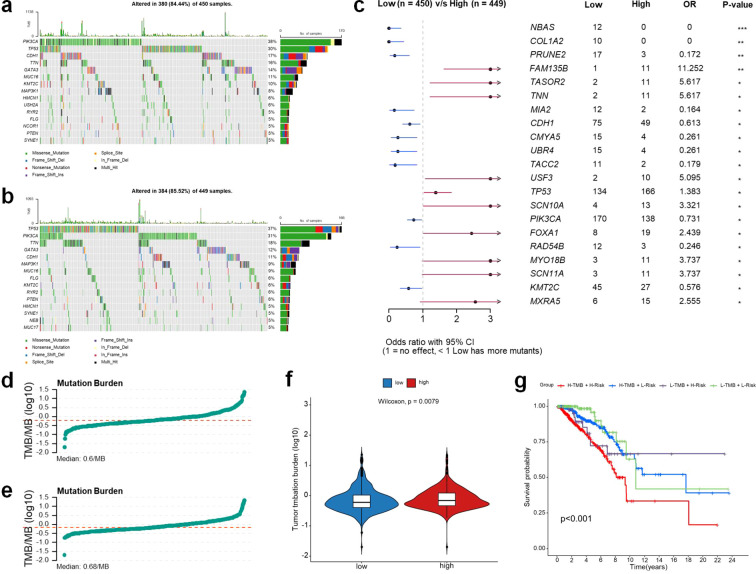
Genomic alterations and TMB analysis between high- and low-risk groups. Mutation landscape of the low-risk **(a)** and high-risk **(b)** groups, showing the frequency and distribution of somatic mutations in BRCA patients. Differentially mutated genes between the low- and high-risk groups **(c)**. TMB distribution in the low-risk **(d)** and high-risk **(e)** groups. Comparison of TMB levels between high- and low-risk groups **(f)**. Kaplan-Meier survival curves comparing OS among patients stratified by both TMB (high vs. low) and risk score (high vs. low) **(g)**. TMB, tumor mutation burden; BRCA, breast invasive carcinoma; OS, overall survival; OR, odds ratio; CI, confidence interval. Statistical significance: *p < 0.05, **p < 0.01, ***p < 0.001, ****p < 0.0001, ns: not significant.

To further investigate the genomic characteristics associated with the snoRNA-based prognostic signature, we analyzed the mutation landscape and tumor mutational burden (TMB) in the high- and low-risk groups. An examination of genomic alterations revealed that the mutation frequency was reduced in the low-risk patients when contrasted with the high-risk patients (84.4% versus 85.52%). Mutations in the TP53 gene were found to be more common within the high-risk group, while mutations in the PIK3CA gene occurred with greater prevalence in the low-risk group (**Figures 6a, b**). Furthermore, the mutation landscape differed significantly between the high- and low-risk groups (**Figure 6c**). Genes such as FAM135B, TAS2R2, TNN, MIA2, and USF3 were more frequently mutated in the high-risk group (OR > 1, p < 0.05), While CDH1, CMYA5, and RAD54B mutations were enriched in the low-risk group (OR < 1, p < 0.05). Beyond mutation profiling, we further evaluated TMB in the high- and low-risk groups defined by the median risk score. TMB was analyzed as a continuous variable and compared between groups using the Wilcoxon rank-sum test (**Figures 6d–f**). Patients were subsequently stratified into four subgroups based on TMB status and risk score for Kaplan–Meier survival analysis (**Figure 6g**).

### Nomogram construction

**Figure 7:**
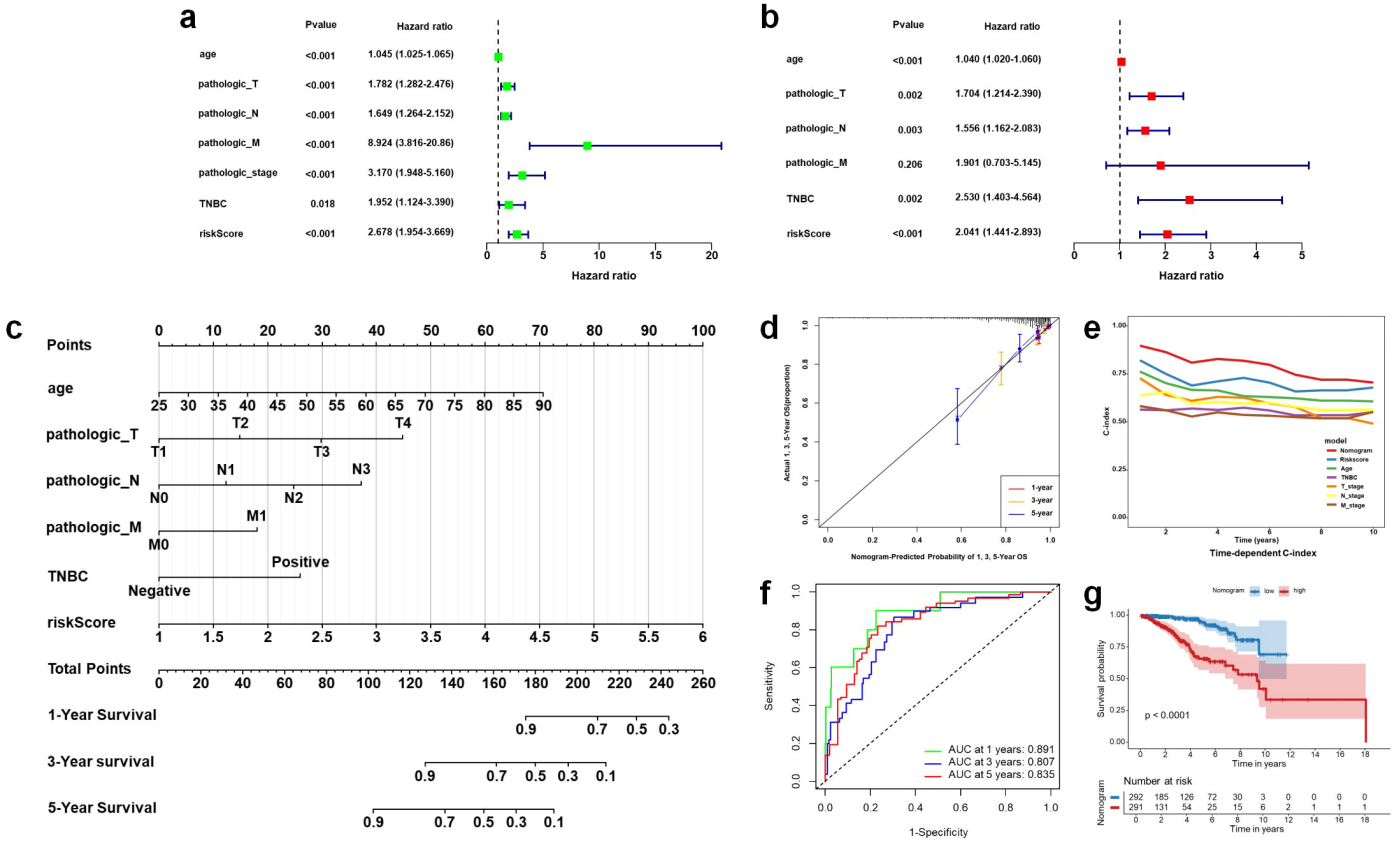
Construction and evaluation of a prognostic nomogram integrating the 8-snoRNA signature and clinical factors. Forest plots of univariate **(a)** and multivariate **(b)** Cox regression analyses for OS in BRCA patients. Nomogram integrating age, pathological T stage, pathological N stage, pathological M stage, TNBC status, and risk score for predicting 1-, 3-, and 5-year OS **(c)**. Calibration curves for the nomogram-predicted 1-, 3-, and 5-year OS **(d)**. Time-dependent C-index curves comparing predictive performance of the nomogram versus individual clinical features **(e)**. Time-dependent ROC curves of the nomogram for 1-, 3-, and 5-year OS prediction **(f)**. Kaplan-Meier survival curves comparing OS between high and low nomogram score groups **(g)**. OS, overall survival; BRCA, breast invasive carcinoma; TNBC, triple negative breast cancer; Concordance index, C-index; ROC, receiver operating characteristic; AUC, area under the curve.

In order to evaluate the independence of our signature as a prognostic factor for patients with BRCA, we conducted both univariate and multivariate Cox regression analyses (illustrated in **Figures 7a, b**). The findings revealed that several variables, including age, pathological T stage, pathological N stage, pathological M stage, overall pathological stage, TNBC, and risk score, exhibited a significant correlation with OS in the univariate analysis. However, multivariate analysis indicated that only age, pathological T stage, pathological N stage, TNBC, and risk score were independent prognostic factors. In order to improve the clinical relevance of our research, we developed a nomogram model founded on critical prognostic indicators, which encompassed age, pathological T stage, pathological N stage, pathological M stage, TNBC, and risk score (**Figure 7c**). The findings indicated that this nomogram demonstrated robust predictive capability for OS at 1-year, 3-year, and 5-year intervals in patients with BRCA (**Figure 7d**). Notably, the C-index of the nomogram surpassed that of the individual predictive factors (**Figure 7e**), underscoring the advantages of this integrated approach. This finding suggests that integrating molecular and clinicopathological variables into a single model can improve individualized prediction of overall survival in patients with BRCA. The AUC values from ROC analysis for predicting OS at 1-year, 3-year, and 5-year intervals were recorded at 0.891, 0.807, and 0.835, respectively (**Figure 7f**), which further reinforces the reliability and clinical relevance of this prognostic nomogram. Furthermore, Kaplan-Meier survival analysis revealed that patients with elevated nomogram scores experienced significantly poorer overall survival outcomes (**Figure 7g**).

### GSEA

**Figure 8:**
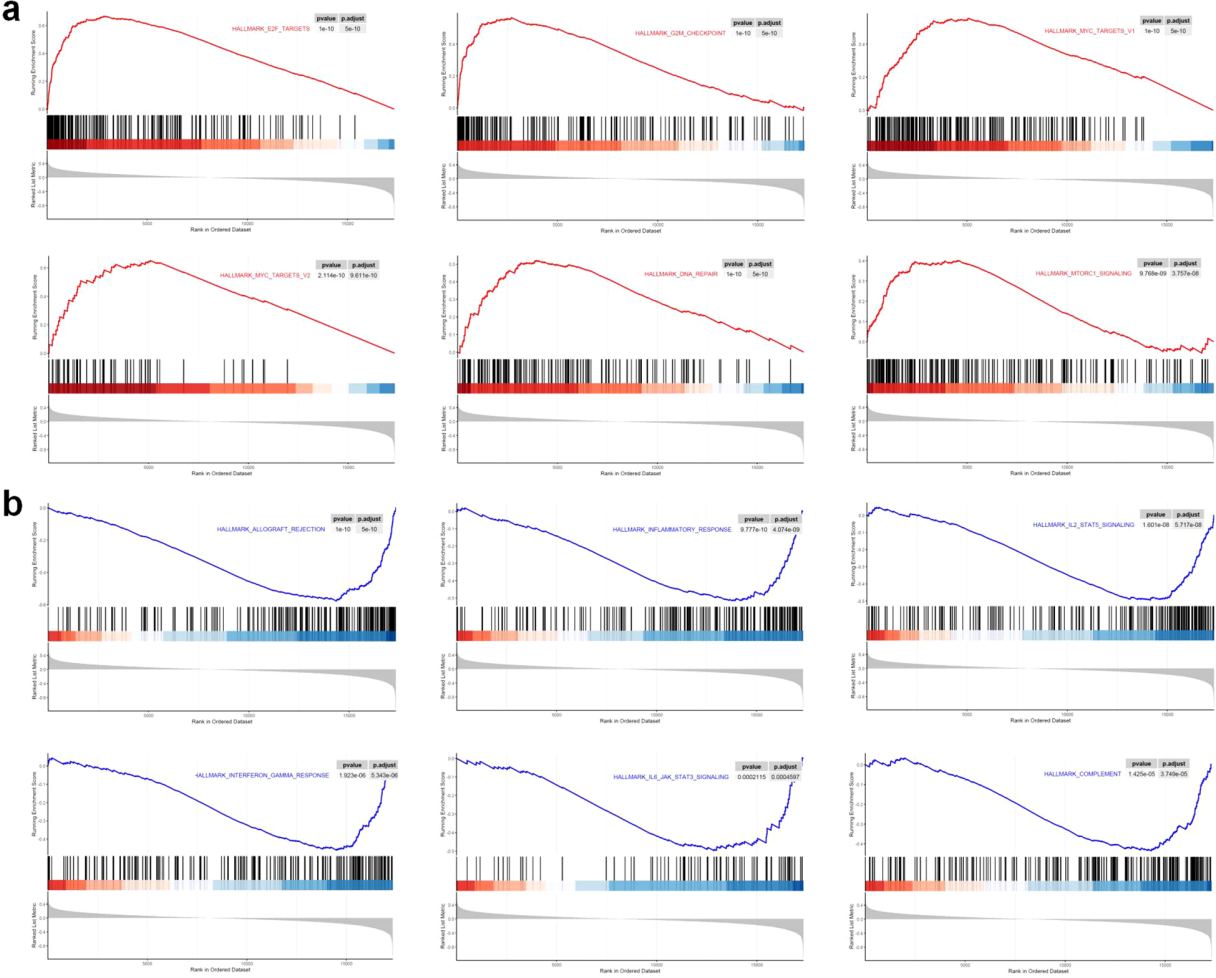
GSEA of high- and low-risk groups based on the 8-snoRNA signature. Significantly enriched hallmark pathways in the high-risk group **(a)**. Significantly downregulated immune-related pathways in the high-risk group **(b)**. GSEA, gene set enrichment analysis.

In order to gain deeper insights into the biological mechanisms that may account for the disparities in survival rates observed between the high-risk and low-risk cohorts as influenced by the 8-snoRNA signature, we performed pathway enrichment analysis using GSEA. As shown in **Figure 8a**, several cancer-related signaling pathways, including E2F targets, G2M checkpoint, MYC targets, DNA repair, and mTORC1 signaling, were significantly enriched in the high-risk group. These pathways are predominantly involved in cell cycle progression and transcriptional regulation, suggesting a heightened proliferative and transcriptional activity in this group. Furthermore, immune-related pathways—including allograft rejection, inflammatory response, IL2-STAT5 signaling, interferon-gamma response, IL6-JAK-STAT3 signaling, and complement pathways—were significantly downregulated in the high-risk group (**Figure 8b**). To provide a global overview of the enriched pathways, the GSEA results of all Hallmarkgene sets are summarized in **Supplementary Figure 2**. These findings suggest a relative reduction in immune-related signaling in the high-risk group, which may contribute to the poorer prognosis observed in patients with elevated risk scores.

### Immune infiltration analysis

**Figure 9:**
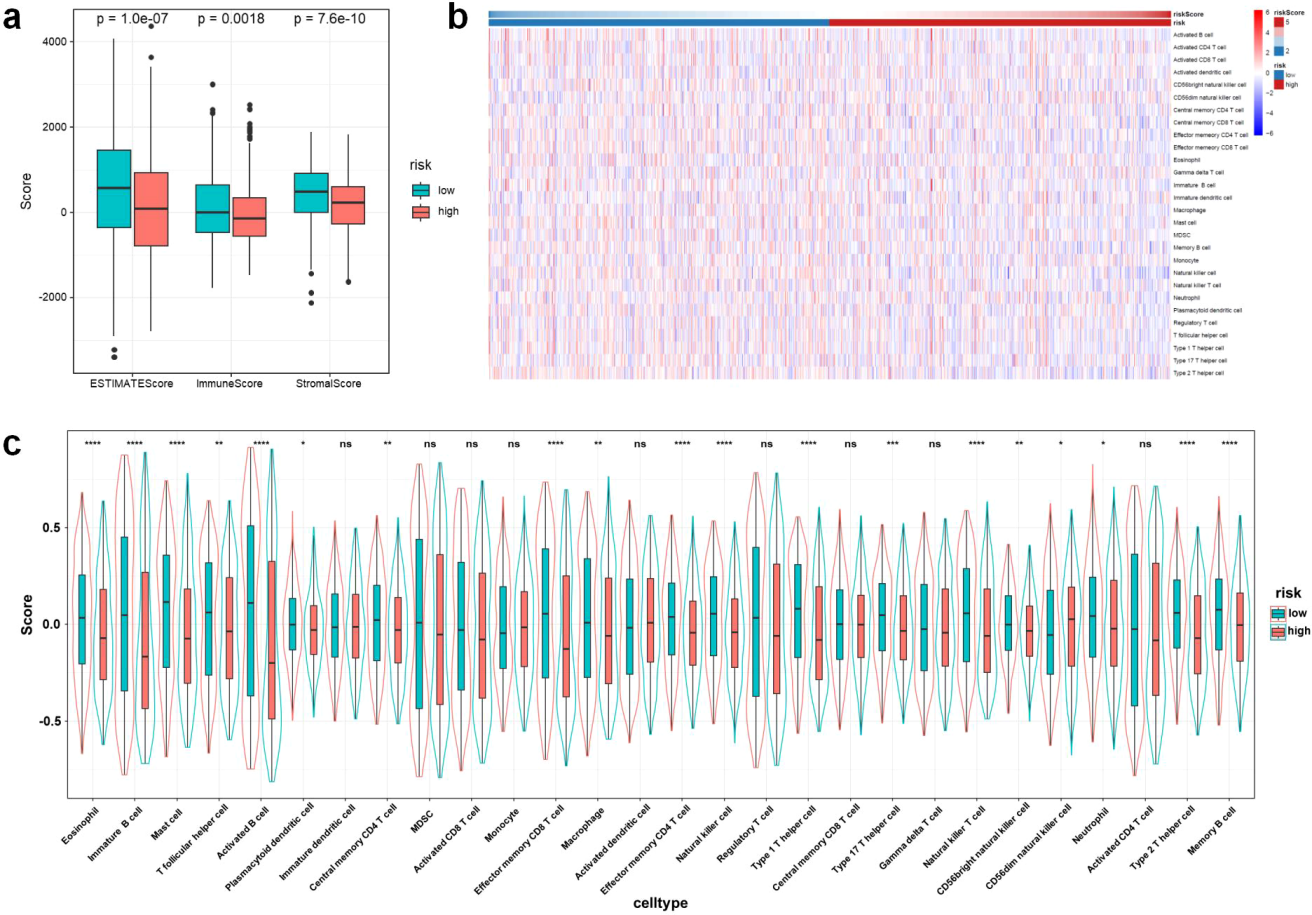
Immune infiltration analysis between high- and low-risk BRCA patient groups. Comparison of ESTIMATE score, immune score, and stromal score between high- and low-risk groups **(a)**. Heatmap showing the relative abundance of tumor-infiltrating immune cells based on ssGSEA in the two groups **(b)**. Violin plots comparing the infiltration levels of 28 immune cell types between high- and low-risk groups **(c)**. BRCA, breast invasive carcinoma; ssGSEA, single-sample gene set enrichment analysis. Statistical significance: *p < 0.05, **p < 0.01, ***p < 0.001, ****p < 0.0001, ns, not significant.

**Figure 10:**
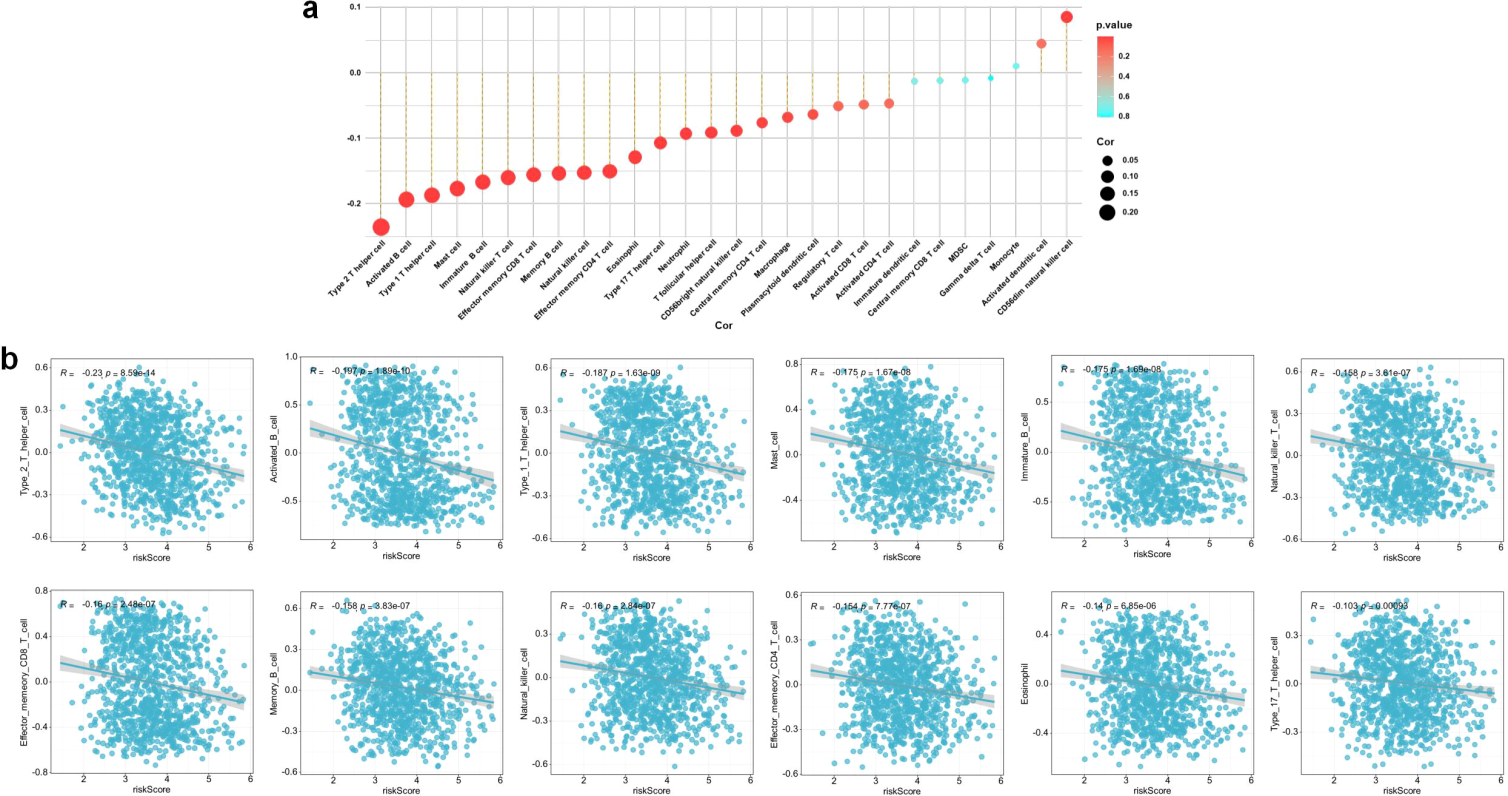
Correlation between the 8-snoRNA risk score and immune cell infiltration. Bubble plot showing the correlation between risk score and the infiltration levels of various immune cell types **(a)**. Scatter plots showing detailed negative correlations between risk score and specific immune cell types **(b)**.

In order to investigate immune infiltration and the tumor immune microenvironment in BRCA, we first evaluated immune scores, stromal scores, and ESTIMATE scores among patient cohorts categorized as high-risk and low-risk. Our results demonstrated that the immune, stromal, and ESTIMATE scores were significantly diminished in patients classified as high-risk (**Figure 9a**). Additionally, we utilized ssGSEA to assess the relationship between risk scores and the enrichment of immune-related signatures. The analysis indicated that the enrichment of most immune cell–related signatures was significantly lower in high-risk patients compared to those in the low-risk category (**Figure 9b**). Specifically, 19 distinct types of immune cells showed markedly reduced levels of infiltration within the high-risk group (**Figure 9c**). Further correlation analysis uncovered a negative relationship between the risk score and most immune cell types (**Figure 10a**). A more granular correlation analysis was subsequently conducted on immune cell types that demonstrated a statistically significant negative correlation with the risk score (cor < -0.1, p < 0.05) (**Figure 10b**). To further corroborate the observed differences in the immune landscape, we employed multi-algorithm immune cell deconvolution techniques, including CIBERSORT, TIMER, XCELL, EPIC, MCPCOUNTER, and QUANTISEQ. These analyses consistently suggested that high-risk patients tended to exhibit reduced estimated levels of anti-tumor immune cell infiltration and increased enrichment of immunosuppressive components, supporting alterations in the tumor immune microenvironment (**Supplementary Figure 1**).

### SNORA11 promotes the proliferation, migration and invasion of BRCA cells

Among the eight snoRNAs constituting our prognostic signature, four (SNORA7B, SCARNA3, SNORA11, and SNORA9) were identified as high-risk factors, showing positive coefficients in the multivariate Cox model and being significantly upregulated in the high-risk group (**Figures 2c, d, g**). Notably, while SNORA7B has been previously reported to function as an oncogene in BC (18), the other three snoRNAs—SCARNA3, SNORA11, and SNORA9—remain largely uncharacterized in this context. To further explore the biological relevance of our model and to elucidate the potential functional role of individual snoRNAs, we selected SNORA11 for experimental validation. This selection was based on its strong association with poor prognosis (HR = 1.36, p = 0.016, **Figure 2c**), its consistent upregulation in high-risk patients (**Figure 2g**), and the absence of prior studies linking SNORA11 to BC, suggesting its potential as a novel oncogenic snoRNA.

**Figure 11:**
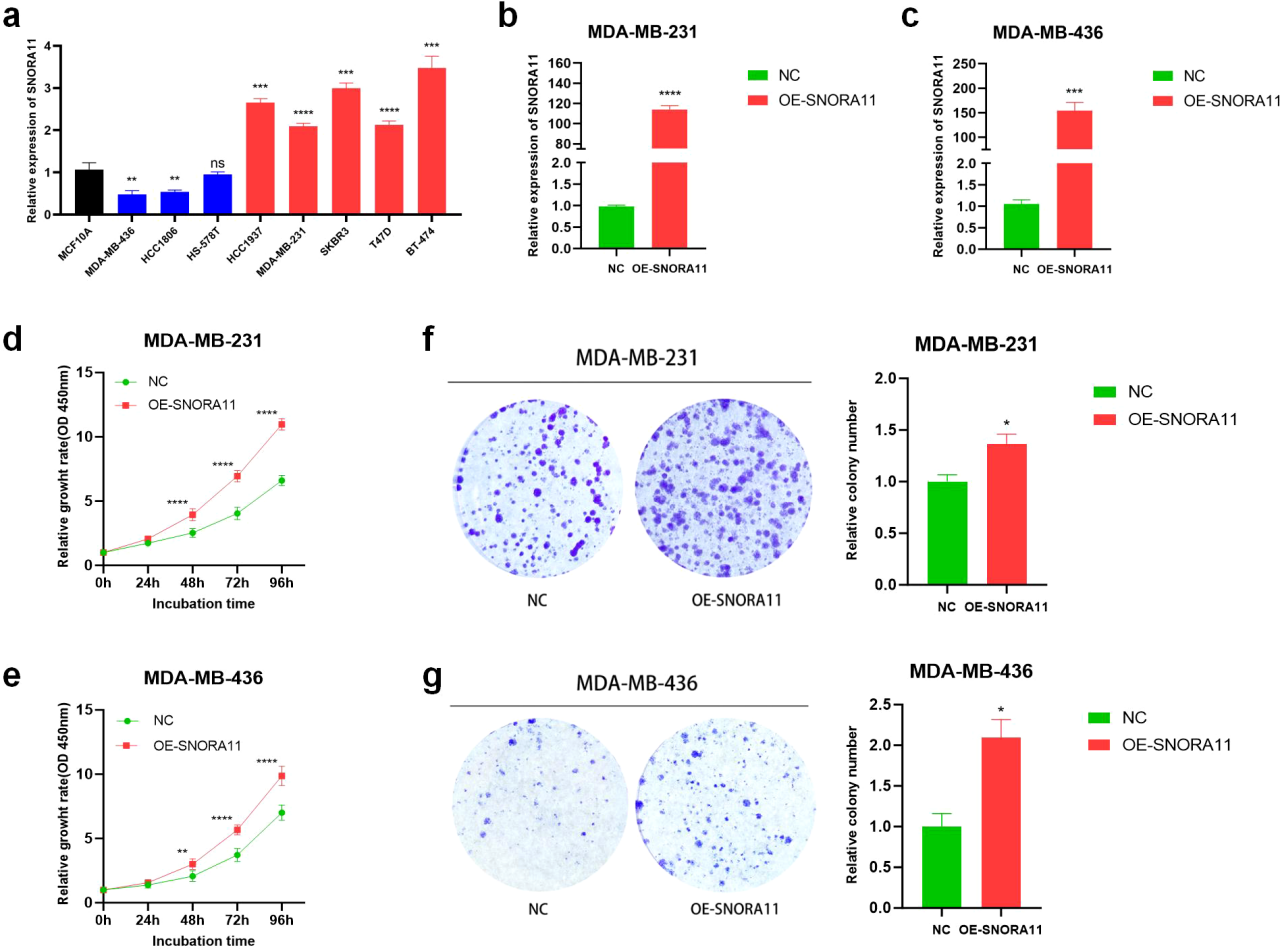
SNORA11 promotes proliferation of BC cells *in vitro.* Relative expression levels of SNORA11 in a panel of BC cell lines and the non-tumorigenic breast epithelial cell line **(a)**. qRT-PCR validation of SNORA11 overexpression in MDA-MB-231 **(b)** and MDA-MB-436 **(c)** cells after plasmid transfection. CCK-8 assays showing enhanced cell proliferation in SNORA11-overexpressed MDA-MB-231 **(d)** and MDA-MB-436 **(e)** cells compared to negative control cells. Colony formation assay assessing the clonogenic potential of MDA-MB-231 **(f)** and MDA-MB-436 **(g)** cells following SNORA11 overexpression. BC, breast cancer; qRT-PCR, quantitative real-time polymerase chain reaction; CCK-8, cell counting kit-8. Statistical significance: *p < 0.05, **p < 0.01, ***p < 0.001, ****p < 0.0001, ns, not significant.

**Figure 12:**
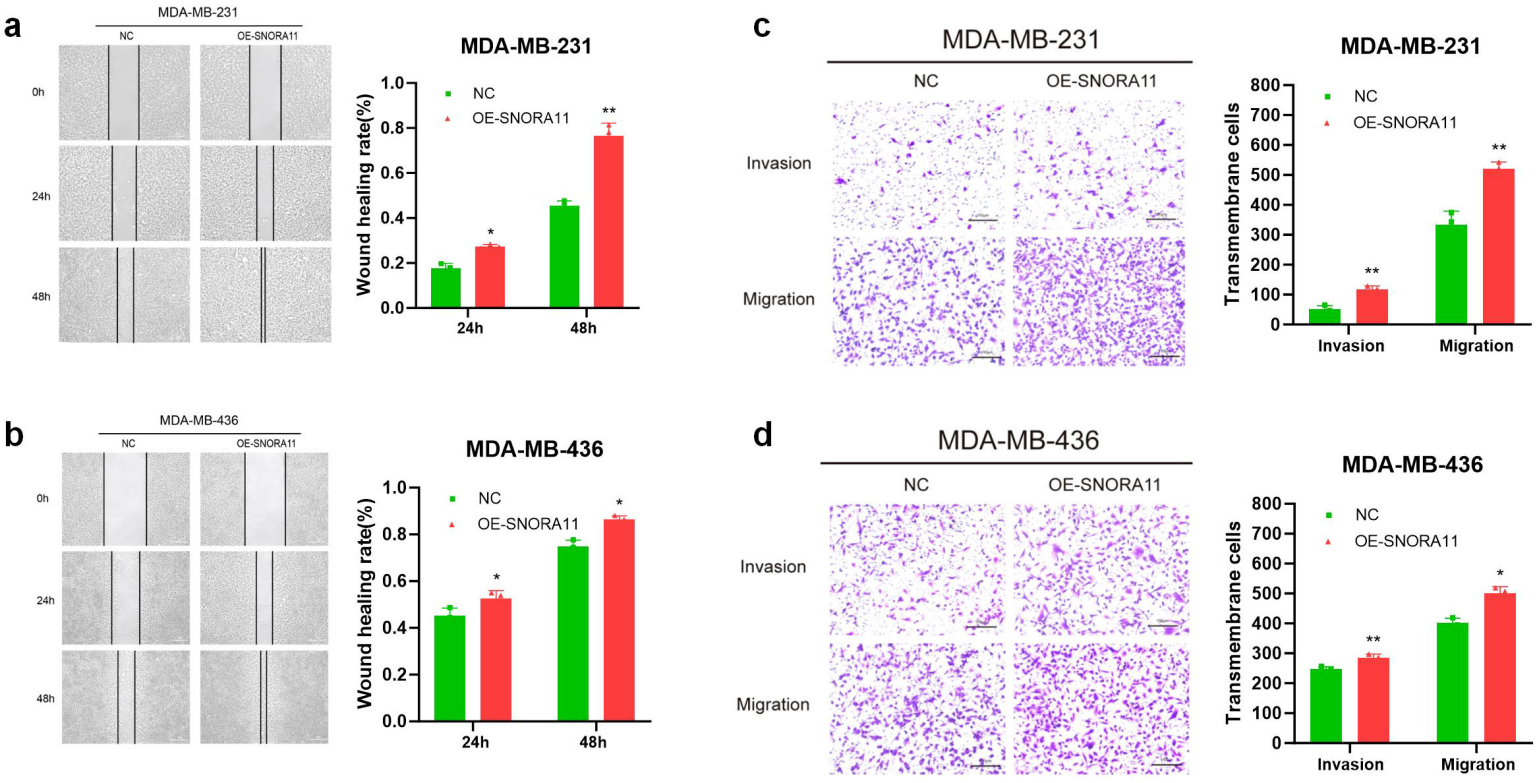
SNORA11 enhances the migration and invasion abilities of BC cells *in vitro.* Wound healing assays showing increased migration rates in SNORA11-overexpressed MDA-MB-231 **(a)** and MDA-MB-436 **(b)** cells at 24 h and 48 h. Transwell assays showing increased migration and invasion rates in SNORA11-overexpressed MDA-MB-231 **(c)** and MDA-MB-436 **(d)** cells. BC, breast cancer. Statistical significance: *p < 0.05, **p < 0.01, ***p < 0.001, ****p < 0.0001, ns, not significant.

To validate the functional role of SNORA11, we first examined its endogenous expression levels across a panel of human BC cell lines and a non-tumorigenic breast epithelial cell line (MCF10A). As shown in **Figure 11a**, the expression of SNORA11 varied across different BC cell lines. Compared with the non-tumorigenic breast epithelial cell line MCF10A, SNORA11 was significantly upregulated in MDA-MB-231, BT-474, T47D, and SKBR3 cells, whereas it was downregulated in MDA-MB-468, HCC1569, and HS578T cells. To further investigate its biological function, we established SNORA11 overexpressed MDA-MB-231 and MDA-MB-436 cell lines via plasmids transfection. Quantitative RT-PCR confirmed robust overexpression of SNORA11 in both cell lines compared to NC cells (**Figures 11b, c**). The CCK-8 assay demonstrated that overexpressing SNORA11 markedly enhanced MDA-MB-231 and MDA-MB-436 cells proliferation (**Figures 11d, e**). The colony formation assay further verified that OE-SNORA11 cells formed significantly more and larger colonies than NC cells in both MDA-MB-231 and MDA-MB-436 lines, further supporting the pro-proliferative role of SNORA11 (**Figures 11f, g**). To assess the impact of SNORA11 on cell motility, wound healing and transwell assays were performed. As shown in **Figures 12a, b**, SNORA11 overexpression significantly enhanced the migration ability of MDA-MB-231 and MDA-MB-436 cells at both 24 h and 48 h. Consistently, transwell assays revealed that SNORA11 overexpression markedly increased both migration and invasion capacities in both cell lines (**Figures 12c, d**). These results suggest that SNORA11 facilitates the migratory and invasive behavior of BC cells.

### Potential mechanisms for SNORA11 to perform biological functions

**Figure 13:**
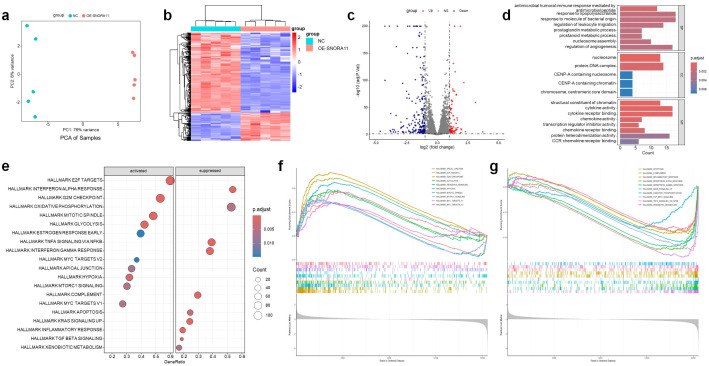
Transcriptomic profiling reveals potential molecular mechanisms of SNORA11 in BC cells PCA showing distinct transcriptomic profiles between SNORA11-overexpressed and control MDA-MB-231 cells **(a)**. Heatmap of DEGs between OE-SNORA11 and NC groups **(b)**. Volcano plot displaying significantly upregulated (red) and downregulated (blue) genes **(c)**. GO enrichment analysis of DEGs **(d)**. GSEA analysis showing hallmark pathways activated or suppressed in the OE-SNORA11 group **(e)**. Representative GSEA enrichment plots of hallmark pathways activated **(f)** or suppressed **(g)** by SNORA11 overexpression. BC, breast cancer; PCA, principal component analysis; DEGs, differentially expressed genes; NC, negative control; GO, Gene Ontology; GSEA, gene set enrichment analysis.

To explore the molecular mechanisms underlying SNORA11-mediated tumor-promoting effects, RNA sequencing was performed on MDA-MB-231 cells overexpressing SNORA11. Principal component analysis (PCA) demonstrated a clear separation between the two groups, indicating distinct transcriptomic profiles (**Figure 13a**). Differential expression analysis identified a substantial number of upregulated (72) and downregulated (202) genes in the OE-SNORA11 group (**Figures 13b, c**), using a cutoff of adjusted p-value < 0.05 and |log_2_FC| > 1. GO enrichment analysis revealed that the differentially expressed genes associated with SNORA11 were significantly enriched in biological processes such as chromatin organization (e.g., nucleosome assembly, structural constituent of chromatin), immune-related functions (e.g., leukocyte migration, cytokine activity, cytokine receptor binding), and transcriptional regulation (e.g., transcription regulator inhibitor activity), suggesting a potential role of SNORA11 in modulating immune responses and chromatin-associated gene regulation (**Figure 13d**). GSEA based on hallmark gene sets further highlighted that SNORA11 overexpression was associated with activation of oncogenic pathways, including E2F targets, G2M checkpoint, MYC targets, and oxidative phosphorylation. Conversely, immune-related pathways such as interferon response, inflammatory response, TNF-α signaling via NF-κB, and complement activation were significantly suppressed (**Figures 13e-g**). These transcriptomic findings suggest that SNORA11 may promote BC progression by enhancing cell cycle and metabolic activity while suppressing immune responses, consistent with its observed phenotypic effects on proliferation, migration, and invasion.

## Discussion

BC remains the most common malignancy and a leading cause of cancer-related death among women worldwide (1, 2). Despite advances in early detection and multimodal therapies—including surgery, endocrine therapy, chemotherapy, targeted agents, and immunotherapy—clinical outcomes remain highly variable (3–5). This heterogeneity stems from diverse molecular subtypes, genetic alterations, and complex tumor microenvironments (6, 7). Current prognostic tools often fail to fully capture disease complexity, limiting their effectiveness in guiding personalized treatment (19, 20). Thus, there is an urgent need for novel biomarkers and robust prognostic models to improve risk stratification and support precision oncology in BC.

SnoRNAs have emerged as important regulators in cancer biology, extending far beyond their traditional roles in rRNA modification (8). Mounting evidence indicates that snoRNAs participate in tumorigenesis by modulating cell proliferation, apoptosis, migration, invasion, and the tumor microenvironment (10, 11, 21–23). These effects are mediated through both canonical functions—such as 2’-O-methylation and pseudouridylation of rRNAs—and noncanonical mechanisms, including regulation of mRNA splicing, chromatin accessibility, and key oncogenic signaling pathways (24–26). In multiple cancer types, including liver, lung, colorectal, and hematologic malignancies, specific snoRNAs have been shown to act as oncogenes or tumor suppressors, and their expression levels correlate with prognosis, metastatic potential, and therapeutic resistance (27–30). In BC, several snoRNAs have been implicated in disease progression and clinical outcomes. For instance, SNORA51 enhances BC stemness via the RPL3/NPM1/c-MYC axis, while SNORD50A/B and U50A modulate apoptosis and drug response through p53 and mTOR pathways, respectively (13, 14, 31). Other snoRNAs, such as SNORA73B, SCARNA4, and SNORD49B, have shown promise as noninvasive diagnostic biomarkers (32). Despite these advances, the functional landscape of snoRNAs in BC remains largely uncharted. Given their oncogenic potential and biomarker relevance, we hypothesized that specific snoRNA expression profiles could serve as effective predictors of prognosis in BC. However, no integrative snoRNA-based prognostic model has yet been established in this context. Moreover, the relationships between snoRNA expression, immune infiltration, molecular subtypes, and treatment response remain poorly defined. Therefore, we aimed to construct a robust snoRNA-based signature that not only stratifies patient risk but also provides insights into tumor biology and immune landscape.

In this study, we systematically constructed a prognostic model based on snoRNA expression profiles in BC using data from the TCGA-BRCA cohort. Through a multi-step approach involving univariate Cox regression, LASSO Cox regression, and multivariate Cox analysis, we identified an 8-snoRNA signature (SNORD114-29, SNORA5A, SNORA54, SNORA7B, SNORA9, SNORA11, SCARNA3, and SNORD64) that effectively stratified patients into high- and low-risk groups, with the high-risk group exhibiting significantly poorer survival outcomes. The model demonstrated robust predictive performance, with AUC values exceeding 0.70 across 1-, 3-, and 5-year time points in both internal validation and entire cohorts, indicating strong temporal stability and accuracy. Furthermore, the risk score showed significant associations with aggressive clinical features, including higher pathological stage, advanced T and N classification, and unfavorable molecular subtypes such as luminal B and basal-like. These findings suggest that the snoRNA-based model not only serves as a reliable prognostic indicator but also reflects underlying tumor biology. Stratified survival analyses confirmed that the model retained prognostic significance across diverse clinical subgroups, including age, TNBC status, and pathological stage. Importantly, integration of the risk score into a multivariable nomogram alongside clinical variables (age, TNM stage, and TNBC status) further enhanced prognostic performance. The resulting nomogram achieved a high concordance index and superior AUCs for 1-, 3-, and 5-year OS (0.891, 0.807, and 0.835, respectively), outperforming individual clinical predictors. Collectively, these results underscore the prognostic value, clinical relevance, and broad applicability of the snoRNA-based model in the personalized management of BC.

To explore the biological mechanisms underlying the survival differences between high- and low-risk groups, we conducted GSEA and immune infiltration profiling. GSEA revealed that high-risk patients exhibited significant enrichment of pathways involved in cell proliferation and transcriptional regulation, including E2F targets, G2M checkpoint, MYC targets, DNA repair, and mTORC1 signaling. These findings suggest that the high-risk group is characterized by heightened proliferative activity and increased genomic instability, which may contribute to more aggressive tumor behavior and poorer clinical outcomes. In contrast, multiple immune-related pathways—such as allograft rejection, interferon-gamma response, IL2-STAT5 signaling, IL6-JAK-STAT3 signaling, and complement activation—were significantly downregulated in the high-risk group. This suppression of immune signaling was further supported by immune microenvironment analysis. High-risk patients exhibited markedly lower immune, stromal, and ESTIMATE scores, indicating reduced immune cell infiltration and stromal content. Single-sample GSEA and multi-algorithm deconvolution consistently suggested decreased estimated infiltration of anti-tumor immune cells (e.g., activated CD8+ T cells, dendritic cells, and NK cells) and enrichment of immunosuppressive components in high-risk tumors. Moreover, the risk score showed a strong negative correlation with the abundance of most immune cell subsets, suggesting alterations in the tumor immune microenvironment. Together, these results suggest that the poor prognosis associated with the high-risk group may be driven by a dual mechanism: enhanced proliferative signaling and impaired anti-tumor immunity. The combination of unchecked tumor cell growth and immune evasion likely contributes to disease progression and resistance to therapy in this subgroup. These findings highlight the potential of the snoRNA signature not only as a prognostic tool but also as a window into the biological behavior of BC, with possible implications for tailoring immunotherapeutic strategies.

Among the eight snoRNAs comprising our prognostic signature, four (SNORA7B, SCARNA3, SNORA11, and SNORA9) were identified as high-risk factors, while the remaining four (SNORD114-29, SNORA5A, SNORA54, and SNORD64) were protective. Several of these snoRNAs have been implicated in tumorigenesis. For instance, SNORA7B, an H/ACA box snoRNA, has been reported to act as an oncogene in both breast and lung cancers by promoting cell proliferation, migration, and invasion through inhibition of apoptosis and regulation of rRNA pseudouridylation (18, 33). Similarly, SNORA5A has been shown to suppress tumor progression in BC by modulating macrophage polarization via the TRAF3IP3 axis, highlighting the dual roles snoRNAs can play as oncogenes or tumor suppressors depending on context (34). However, the biological functions of other snoRNAs in our model, including SCARNA3, SNORA9, and especially SNORA11, remain largely unexplored in BC or other malignancies.

To investigate the functional contribution of individual model components, we selected SNORA11 for *in vitro* validation based on several compelling factors: its strong association with poor prognosis (HR = 1.36, p = 0.016), consistent upregulation in high-risk patients, and lack of prior characterization in BC. Expression profiling confirmed that SNORA11 is upregulated in multiple BC cell lines compared to non-tumorigenic epithelial cells. Functional assays revealed that overexpression of SNORA11 significantly enhanced proliferation, colony formation, migration, and invasion in MDA-MB-231 and MDA-MB-436 cell lines, supporting its oncogenic potential. These findings suggest that SNORA11 may contribute to the aggressive phenotype observed in high-risk patients and highlight its potential as both a prognostic marker and therapeutic target. Notably, SNORA11 was also identified in a recent hepatocellular carcinoma study as a component of a diagnostic and prognostic snoRNA panel, further supporting its cross-cancer relevance (35). Collectively, our results provide the first experimental evidence for the oncogenic role of SNORA11 in BC and underscore the biological validity of our snoRNA-based prognostic model.

To elucidate the molecular mechanisms underlying SNORA11-mediated effects, we performed transcriptome sequencing on SNORA11-overexpressing MDA-MB-231 cells. Principal component analysis revealed distinct transcriptomic profiles between SNORA11-overexpressing and control cells, indicating broad regulatory impact. Differential expression analysis identified over 270 dysregulated genes, and subsequent GO enrichment analysis highlighted significant involvement in chromatin organization, transcriptional repression, and immune-related processes, including cytokine activity and leukocyte migration. These findings suggest that SNORA11 may modulate both epigenetic regulation and the tumor immune microenvironment, consistent with previous reports on the roles of snoRNAs in chromatin remodeling and immune modulation (36–39). Furthermore, GSEA based on hallmark gene sets revealed activation of proliferative and metabolic pathways, such as E2F targets, G2M checkpoint, MYC targets, and oxidative phosphorylation, alongside suppression of immune-related pathways including interferon response, TNF-α signaling via NF-κB, and complement activation. These transcriptomic alterations are consistent with the observed phenotypic effects of SNORA11, suggesting that it may promote BC progression by enhancing cell cycle activity and metabolic output while concurrently dampening anti-tumor immune responses. This dual role may partially explain its prognostic significance in our model and warrants further mechanistic investigation.

Despite the promising findings of our study, several limitations should be acknowledged. First, the prognostic model was developed and validated using retrospective data from the TCGA cohort, including the training, testing, and entire cohorts, which represent internal validation only. As a result, this study is inherently limited by its retrospective design. Due to the lack of publicly available BC datasets with compatible snoRNA expression profiles and complete survival information, independent external validation was not performed in this study. Therefore, validation in independent, multi-center, and prospective cohorts will be necessary to further confirm the generalizability and clinical utility of the proposed prognostic model. Second, although we identified SNORA11 as a potential oncogenic snoRNA and validated its functional role *in vitro*, the functional experiments in this study were primarily based on overexpression assays. Attempts to silence SNORA11 using conventional siRNA or shRNA approaches were not successful, likely due to the short length and structural characteristics of snoRNAs. Therefore, loss-of-function experiments and *in vivo* validation were not performed in the present study and will be necessary to further confirm the biological role of SNORA11 in breast cancer. In future studies, alternative strategies such as antisense oligonucleotides (ASOs) or CRISPR/Cas9-based genome editing will be explored to achieve effective suppression of SNORA11 and to further investigate its downstream molecular mechanisms. In addition, the broader mechanistic landscape of how SNORA11 and other model-included snoRNAs regulate tumor progression remains incompletely understood, and the downstream targets, interacting partners, and potential molecular mechanisms of SNORA11 warrant further investigation. Third, our immune infiltration analysis was based on computational deconvolution algorithms and transcriptomic data, which may not fully capture the dynamic and spatial complexity of the tumor immune microenvironment; future studies incorporating single-cell RNA sequencing or spatial transcriptomics could provide deeper insights. Addressing these limitations in future studies will be essential to fully translate snoRNA-based biomarkers into clinical applications and to better understand their roles in BC biology.

## Conclusions

In this study, we developed and validated a robust 8-snoRNA-based prognostic signature for BC using TCGA transcriptomic and clinical data. The model effectively stratified patients into high- and low-risk groups with significant differences in overall survival and was associated with aggressive clinicopathological features. Functional enrichment and immune infiltration analyses revealed that high-risk patients exhibited enhanced proliferative signaling and suppressed anti-tumor immune responses, suggesting a mechanistic basis for poor prognosis. Experimental validation identified SNORA11 as a novel oncogenic snoRNA that promotes BC cell proliferation, migration, and invasion *in vitro*. Transcriptome analysis further revealed that SNORA11 overexpression is associated with enhanced proliferative signaling and suppressed immune-related pathways, mirroring the molecular features of the high-risk group defined by our prognostic model. Collectively, our findings highlight the prognostic and biological relevance of snoRNAs in BC and provide a foundation for future studies investigating their utility as biomarkers and therapeutic targets.

## Data Availability

The original contributions presented in the study are included in the article/**Supplementary Material**. Further inquiries can be directed to the corresponding author.
