# Supplementary figures and images for: Integrative analysis of a novel snoRNA-based prognostic signature in patients with breast cancer
Source: Front Oncol. 2026 Apr 13;16:1779697. doi: 10.3389/fonc.2026.1779697 (PMC13112537; doi:10.3389/fonc.2026.1779697)

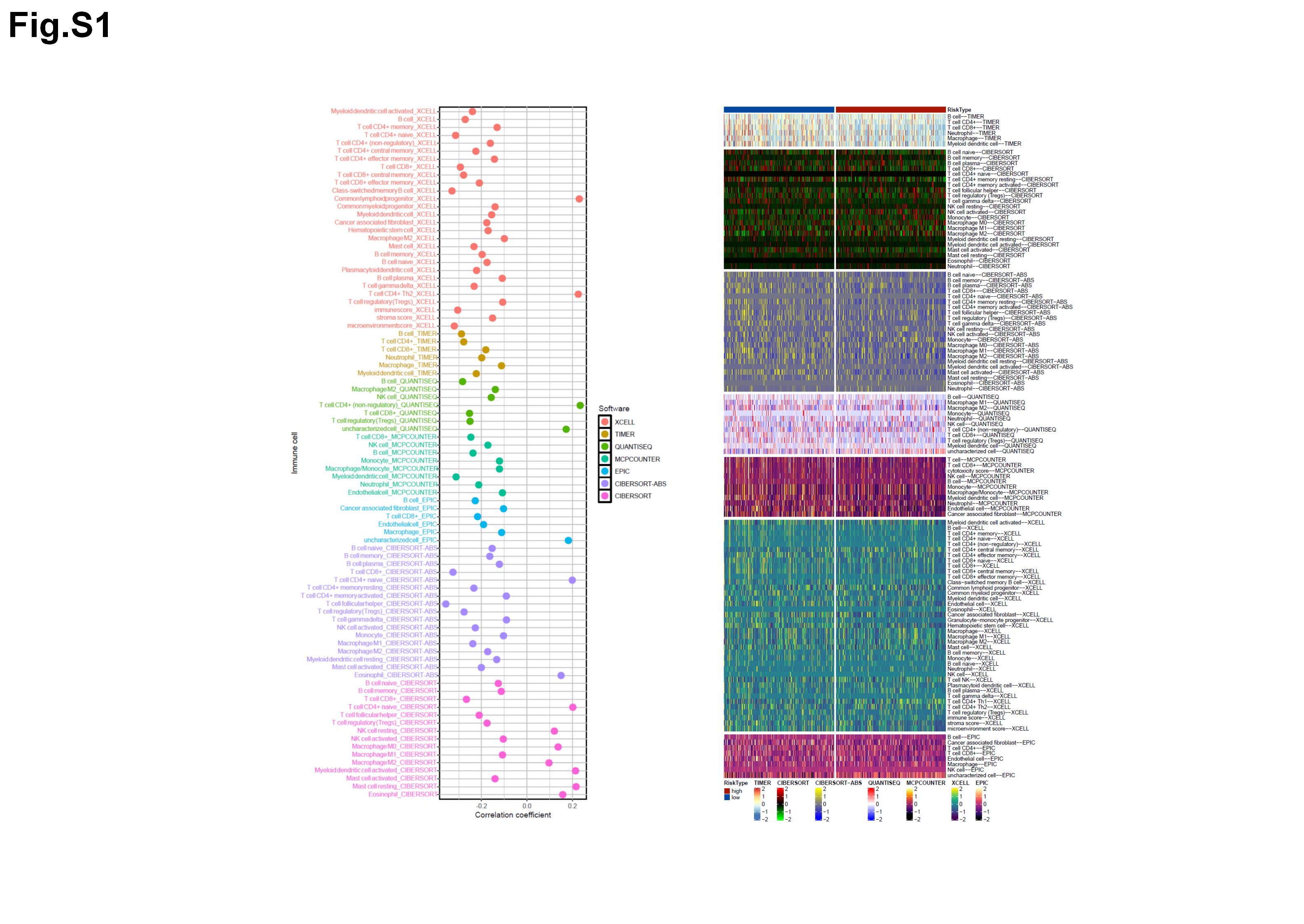

Supplement: Supplementary file 1 [file Image1.jpeg]

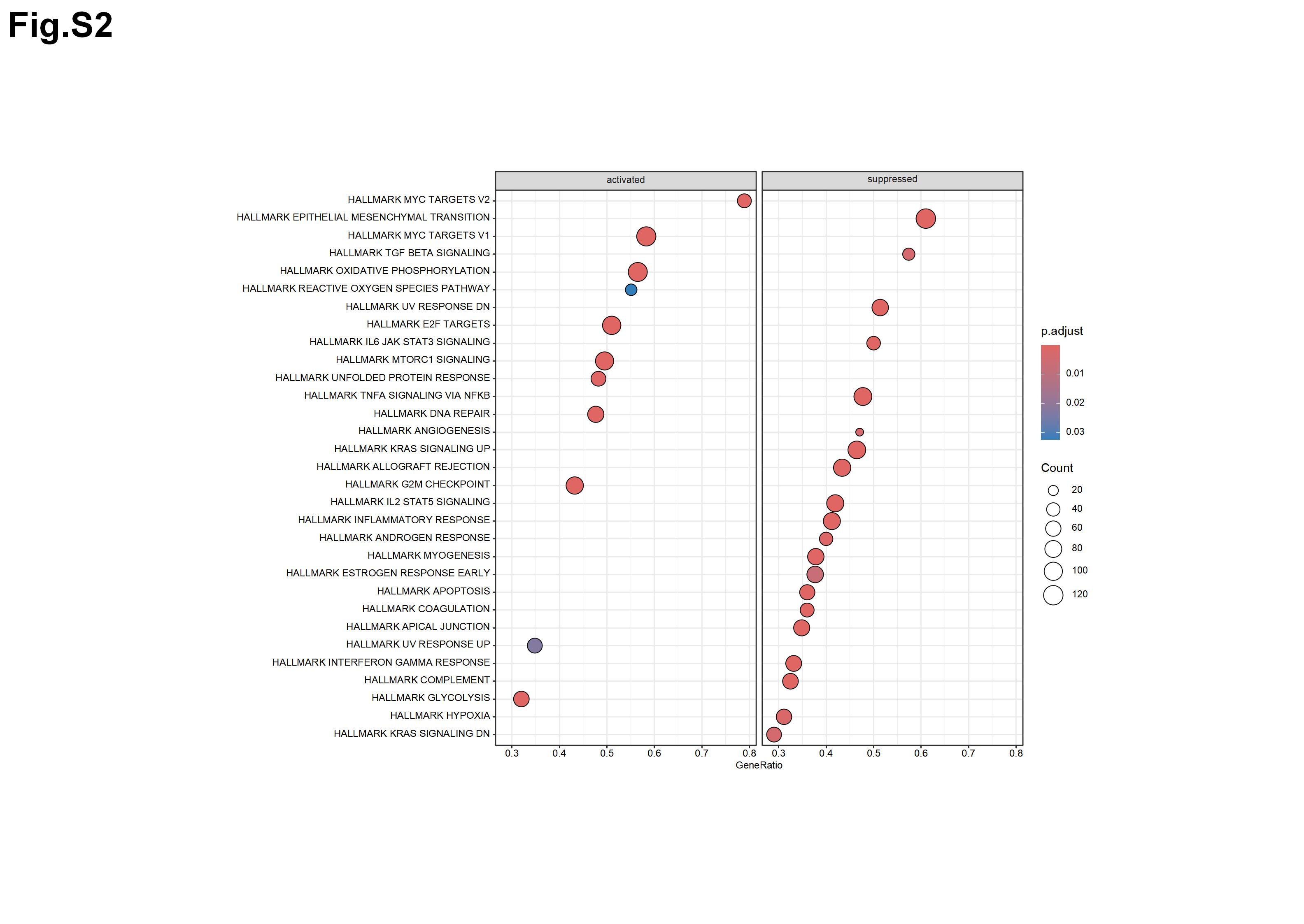

Supplement: Supplementary file 2 [file Image2.jpeg]
